# Synthesis of homo- and hetero-metallic cobalt and zinc nano oxide particles by a calcination process using coordination compounds: their characterization, DFT calculations and capacitance behavioural study†

**DOI:** 10.1039/d0ra01191f

**Published:** 2020-04-01

**Authors:** Sartaj Tabassum, Mohammad Usman, Hamad A. Al-Lohedan, Mahmood M. S. Abdullah, Mohamed A. Ghanem, Merfat S. Al-Sharif, Mohd Sajid Ali

**Affiliations:** Department of Chemistry, Aligarh Muslim University Aligarh-202002 India tsartaj62@yahoo.com +919358255791; Department of Chemistry, College of Sciences, King Saud University P.O. Box 2455 Riyadh 11451 Kingdom of Saudi Arabia

## Abstract

Nano cobalt and porous zinc–cobalt oxide particles were synthesized using the concept of coordination compounds of the type [M(ii)L,L′] (where M(ii) = Co(ii) & Zn(ii) L= 4-hydroxy benzaldehyde and L′ = piperazine) and were thoroughly characterized. Because the precursors are coordination compounds possessing specific geometry in the crystal lattice, uniform and appropriately sized homo- and heterometallic nanocrystals of Co_3_O_4_ and ZnO·Co_3_O_4_ were obtained after a thermal process. The homo and hetero composite particles were characterized by transmission electron microscopy (TEM), scanning electron microscopy (SEM), energy dispersive X-ray analysis (EDX), X-ray diffraction (XRD), FT IR spectroscopy and electrochemistry. The paramagnetic chemical shift of the methyl protons in DMSO due to the nanoparticles was studied by NMR spectroscopy, which indicated that the cobalt particles were ferromagnetic. The structural design modification and surface area of Co_3_O_4_ was improved by adding the ZnO component. DFT calculations were done to validate the nano structure. Supercapacitance ability of the nanoparticles was studied by cyclic voltammetry, and electrochemical calculations were performed to determine the microelectronic characteristics of the material. The specific capacitance was estimated at 207.3 and 51.1 F g^−1^ for the ZnO·Co_3_O_4_ and Co_3_O_4_ electrodes, respectively. Clearly, ZnO·Co_3_O_4_ exhibited a much higher specific capacitance than the Co_3_O_4_ nanocrystal, which was attributed to better conductivity and higher surface area. The capacitance activity showed multifold enhancement due to the porous nature of Zn oxide in the heterometallic nano ZnO·Co_3_O_4_ composite.

## Abstract 2

Pictorial depiction of appropriately sized homo and hetero nanocrystals of Co_3_O_4_ and ZnO·Co_3_O_4_ and the optimized structures of [Co_3_O_4_]_4_ [ZnO]_4_ DMSO adduct.

## Graphical abstract

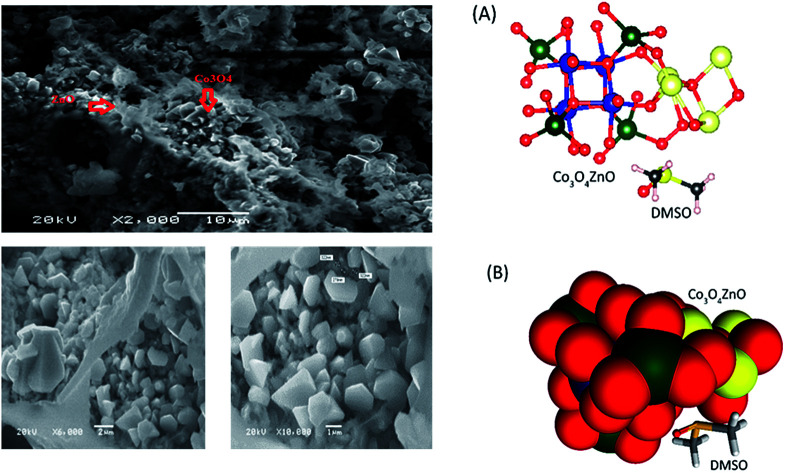

## 1. Introduction

Transition metal oxides have gained considerable interest in recent years owing to their interesting magnetic, optical field emission and biomedical applications. Transition metal ions exist in variable oxidation states, which make them versatile precursor materials for use in nanoscale chemistry. Among the prominent mixed valence oxides, spinel type cobalt oxides (Co_3_O_4_) have attracted considerable attention in the areas of environmental science, catalysis and medicine. For example, many Co_3_O_4_ NPs have been utilized for the degradation of pollutants and in electro catalysis for oxygen hydrogen generation, and very recently, cobalt nano oxides were used as probes in medical diagnostic devices.^1–4^ A glucose sensor of cobalt oxide nano rods was prepared by Kuo-Chuan Ho *et al.* for the non-enzymatic detection of glucose.^5^ The interesting applications of nano transition oxides depend on their size and different structural morphologies including nanotubes, nanorods, nano cubes and meso porous structures.

Many synthetic techniques and routes have been utilized to prepare these nanomaterials, including sol–gel methods,^6^ solvothermal synthesis,^7^ thermal decomposition of cobalt precursors,^8^ sonochemical methods,^9^ co-precipitation^10^ and microwave-assisted methods.^11^ Most of these methods are not feasible for large-scale production owing to the expensive and toxic chemicals required and the use of complex instruments. Researchers are looking for more facile synthetic routes to obtain new nanomaterials of mixed valence oxides by choosing appropriate precursors, which have potential advantages including high yield of pure products, the absence of solvents, low energy consumption and functional efficiency. Herein, we have undertaken the task of preparing homo and hetero-metallic Co_3_O_4_, ZnO·Co_3_O_4_ mixed-valence oxides possessing different structural morphologies and electrochemical behaviour.^12,13^ In this work, we report a new modified calcination process that uses the coordination chemistry concept of employing piperazine and aldehyde with metal salts to obtain a uniform single crop of nano Co_3_O_4_ and porous ZnO·Co_3_O_4_ crystals. The heterobimetallic oxides show multifold enhanced activities (catalytic and capacitance properties) compared to monometallic nano oxide.^14^ The obtained heterobimetallic nanomaterials have mixed oxidation sates, which helps build up the inner electric field at the junction interface and create more pores in the porous material.^15,16^

## 2. Experimental

### 2.1. Materials

Chemicals, including CoCl_2_, Zn(NO_3_)_2_·6H_2_O (4-hydroxy benzaldehyde), and piperazine were purchased from Sigma Aldrich, USA. Power X-ray diffraction (XRD) of the products was measured using a Philips X'Pert PRO MPD diffractometer at a scanning rate of 4° min^−1^, with 2*α* ranging from 10° to 70°, using Cu Kα radiation (=1.5406 Å). The morphologies of the samples were studied by scanning electron microscopy (SEM) (JEOL SM5600LV) at 20 kV. The powders were ultrasonicated in ethanol, and a drop of the suspension was dried on a carbon-coated microgrid. Transmission electron microscopy (TEM) observations were performed with a JEM 100CX-II microscope operated at 100 kV. NMR spectra were recorded in DMSO d_6_ on a Jeol 400 MHz NMR spectrometer. Thermal studies were performed using a TGA/SDTA 851e (Mettler Toledo) thermogravimetric analyser in ambient atmosphere from 20 °C to 700 °C at a heating rate of 10 °C min^−1^. Electrochemical measurements were performed using a potentiostat (AutolabPGSTAT101) in a standard three-electrode setup, with a working electrode of CoZnO_2_ and Co_3_O_4_ nanocrystals (50 μg dispersed in water and isopropanol solution) loaded on a carbon paper substrate (SIGRACET®, grade GDL-24BC, geometric area 1 × 1 cm^2^), as well as a Pt mesh and a saturated calomel electrode (SCE) as the counter and reference electrodes, respectively.

### 2.2. Synthesis of precursors and nanoparticles

**Scheme 1:**
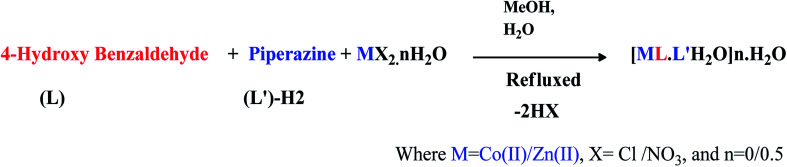
Synthetic route of homo and heterometallic nano particles.

The mono and heterobimetallic nanoparticles were prepared by a modified coordination chemistry procedure. A methanolic solution of the metal salt Co(ii)/Zn(ii), aldehyde and piperazine at a 1 : 1 : 1 molar ratio was refluxed for 2 h in a 100 mL round bottom flask (Scheme 1). The blue cobalt complex and white powder of the zinc compound were obtained, and adducts were washed with methanol and hexane and were dried under vacuum. The prepared coordination compounds were characterized by FTIR and mass spectrometry on the basis of the preliminary characterization (Scheme 1). [Co(ii)benzaldehyde·piperazine·H_2_O]0.5H_2_O; mp 300 °C d*m*/*z* 292.15(293.20) [M–L–L′1.5H_2_O + H]. [Zn(ii)benzaldehyde·piperazine·H_2_O]; mp 235 °C d*m*/*z* 289.65(293.19) [Zn–L·L′·H_2_O–3H^+^]. The FTIR bands at 498, 877, 998, 1224, 1342, 1413, 1450, 1584, 2825, 3196 and 3556 cm^−1^ are due to the C=O, C–C, C–H, C–N and H_2_O vibration and bending modes. The broad bands in the 3556–3196 cm^−1^ range in the spectra of the precursors have been attributed to the stretching vibrations of H_2_O, OH and NH. The band at 2825 cm^−1^ was due to the C–H stretching mode, and the bands at 1342 and 1584 cm^−1^ were assigned to the bonding of M(ii) with N–H and C–O. Another band due to C–O stretching was observed at 1224 cm^−1^ in the spectra of the precursors. The low-frequency absorptions at 498 and 877 cm^−1^ were attributed to the M–O stretching and M–O bending vibrations in the spectra of the complexes.^17^ The [Co(ii)benzaldehyde·piperazine·H_2_O]0.5H_2_O was homogenized by ultrasonication. The powder was thoroughly washed with anhydrous ethanol to remove impurities. The compound was dried in an air oven at 60 °C for 12 h and then calcinated at 500–600 °C for 6 h in an electric furnace. Co_3_O_4_ nanoparticles were prepared with 0.75 g of the complex [Co(ii)benzaldehyde·piperazine·H_2_O] in a porcelain crucible and placed in the furnace. The compound was heated to 500 °C at a rate of 10 °C min^−1^. The cobalt nanoparticles were characterized by various spectroscopic methods. The heterometallic nanocomposite was formed by mixing of [Co(ii)benzaldehyde·piperazine·H_2_O]0.5H_2_O and [Zn(ii)benzaldehyde·piperazine·H_2_O], coordination compounds at a 1 : 5 ratio. The high concentration of the zinc coordination compound in methanol was used because Zn forms a porous material; hence, pockets of Co_3_O_4_ will be formed, which will increase the surface area in the ZnO·Co_3_O_4_ composite material. The mixture was sonicated for approximately 25 minutes in methanol, filtered, washed with hexane and dried in an oven at 60 °C. The nanoconjugate was prepared by calcination, as reported for homometallic Co_3_O_4_.

### 2.3. Computational method

We carried out a series of theoretical calculations of ZnO·Co_3_O_4_ molecular aggregates in order to find out how closely they could approach each other and which atoms and their interactions would be involved in the adduct formation. Herein we adopt a three step molecular modeling protocol, (1) generation of Co_3_O_4_ and ZnO nano particles molecular coordinates from the X-ray crystal structures in two different sizes *e.g.* [Co_3_O_4_]_4_ (9.00 nm), [Co_3_O_4_]_10_ (12 nm), [ZnO]_4_ (5 nm), and [ZnO]_20_ (10 nm). (2) Geometry optimization of [Co_3_O_4_]_*n*_, *n* = 4, 10 with [ZnO]_*n*_, *n* = 4, 20, by employing molecular mechanics force field which includes van der Waals (Lennard-Jones potential), hydrogen bonding, desolvation and electrostatic terms and treats the intramolecular bonds and bond angles of both the molecules as rigid. (3) DFT calculation of three geometrically optimized [Co_3_O_4_]_*n*_·[ZnO]_*m*_ adducts (where *n*: 4, 10 and *m*: 4, 20), (a) [Co_3_O_4_]_4_ [ZnO]_4_, (b) [Co_3_O_4_]_10_ [ZnO]_4_, and (c) [Co_3_O_4_]_10_ [ZnO]_20_. All the molecular mechanic energy-minimization of corresponding molecular adducts were done using Autodock 4.2 software.^18^ All reported DFT computations were performed using ORCA computational package^19^ for previously optimized structures. The single point energy calculation carried out by unrestricted B3LYP functional^20^ using Aldrich's def2-TZVP basis set for all the atoms^21^ to calculate the HOMO and LUMO energies. To speed up the calculations we have used the resolution of identity (RI) approximation with the decontracted auxiliary def2-TZV/J Coulomb fitting basis sets and the chain-of-spheres (RIJCOSX) approximation to exact exchange as executed in ORCA. DFT calculation utilizes the atom-pairwise dispersion correction with the Becke–Johnson damping scheme (D3BJ).^22^

## 3. Results and discussion

The precursors of homo- and heterometallic nano oxides were prepared by using the coordination chemistry concept with a low-cost starting material. The characterization and morphology of the nano Co_3_O_4_ and ZnO·Co_3_O_4_ were studied by TEM and SEM images. The TEM images showed that Co_3_O_4_ and ZnO·Co_3_O_4_ particles possessed a rectangular unit cell with several vertices of Co_3_O_4_ (size 19.14–56.20 nm) and porous ZnO crystalline (22.79 nm pore size) pockets filled with Co_3_O_4_. The particle size of the rectangular pyramidal units was 14.14–16.13 nm. On the basis of the TEM images, it can be concluded that particle overlap occurred and that different long nanoconjugates were obtained by interfacial reactions and agglomeration. The SEM results corroborated well with the TEM results for the Co_3_O_4_ and ZnO·Co_3_O_4_ nanoparticles. This procedure is important because we can obtain identical nano oxide particles with a defined geometry by repeating the procedure.

### 3.1. Infrared spectroscopy

FTIR spectra were recorded for cobalt and zinc nanoparticles to confirm the bonding of oxides to metal ions in the nanocrystal materials and the cationic position in the structure. The FTIR spectra (Fig. S1 and S2†) showed two characteristic stretching vibrations bands of the M–O bonds in Co_3_O_4_. A sharp band appeared at 583 cm^−1^ due to the vibration of Co(iii) ions in the octahedral void of oxide ions. The second band appeared at 665 cm^−1^, confirming the presence of Co^2+^ in the tetrahedral hole, which resulted in the formation of pure nanocrystals.^23^ A similar FTIR was obtained for ZnO·Co_3_O_4_, with additional weak bands at a lower frequency of 580 cm^−1^, which indicated the presence of Zn oxide with Co_3_O_4_.^24^ The cobalt was present as Co^2+^, and two cobalt ions were in the Co^3+^ oxidation state. Mixed oxidation states (divalent and trivalent ions) provide crystal field stabilization at the octahedral (Co^3+^) and tetrahedral (Co^2+^) sites of Co_3_O_4_. No other band was observed, thus confirming the purity of the oxide nanoparticles.

### 3.2. Thermo gravimetric analysis (TGA)

The TGA analysis of the [M(ii)benzaldehyde·piperazine·H_2_O]*n*H_2_O, with M = Co(ii)/Zn(ii), indicated a double-step weight loss between 573–763 K. The weight loss indicated the decomposition of [M(ii)benzaldehyde·piperazine·H_2_O] into the metal oxides Co_3_O_4_/ZnO. The weight loss was found to be approximately 68%, which is equivalent to the loss of water, aldehyde and piperazine compounds to form cobalt oxide/zinc oxide. The observed weight loss of cobalt oxide was close to the theoretical value. Upon the calcination of the obtained [Co(ii)benzaldehyde·piperazine·H_2_O]*n*H_2_O and the mixture of [Co(ii)benzaldehyde·piperazine·H_2_O]*n*H_2_O, [Zn(ii)benzaldehyde·piperazine·H_2_O] at 773 K in air, both metal complexes were converted into Co_3_O_4_ and ZnO·Co_3_O_4_ nanoparticles. The TGA (Fig. S3†) curves of the Co_3_O_4_ oxide nanoparticles show no weight loss, indicating the purity and stability of the particles. The XRD pattern of the sample also clearly indicated that nano Co_3_O_4_ particles are pure oxides.

### 3.3. XRD measurement

**Fig. 1:**
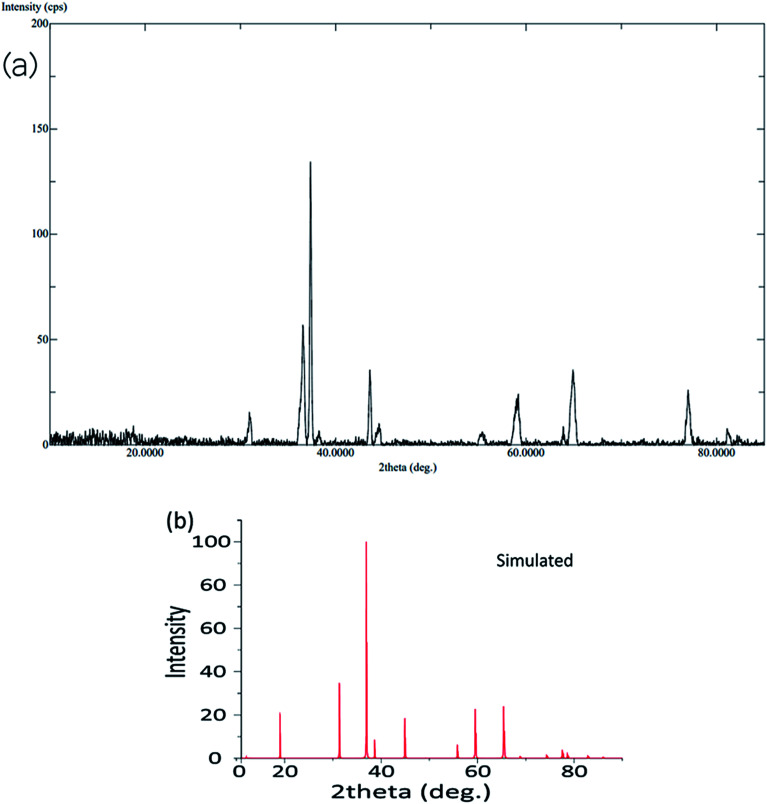
Experimental and simulated XRD pattern of Co_3_O_4_ nanoparticles.

**Fig. 2:**
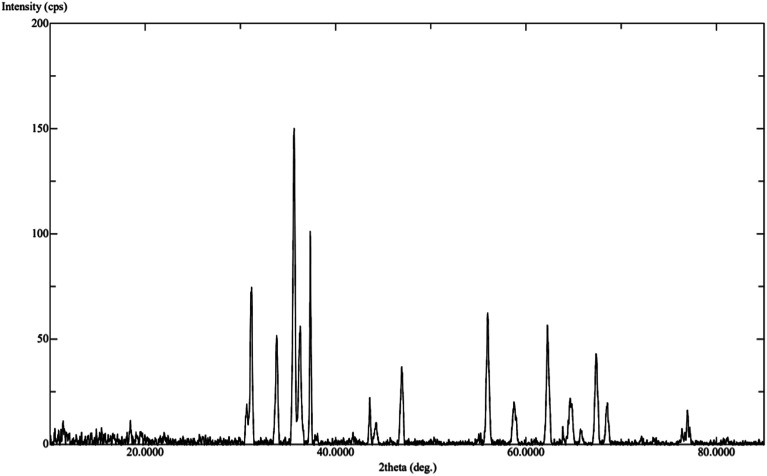
XRD pattern of ZnO·Co_3_O_4_ nanoparticles.

The calcination process was performed directly at 500–600 °C to convert the mono metallic compound and mixture of two complexes to get homo and heterometallic oxides. The nano Co_3_O_4_ and ZnO·Co_3_O_4_ products maintained the crystalline phases of the oxides over time (Fig. S4†). The X-ray diffraction confirmed that both the nano Co_3_O_4_ and ZnO·Co_3_O_4_ composite were pure. The peaks of the Co_3_O_4_ and ZnO·Co_3_O_4_ were observed at 2*θ* = 36.56°, 37.56°, 38.24°, 43.58°, 64.92°, and 65.04° due to the 111, 220, 311, 400, 440 and 511 planes for Co_3_O_4_. For ZnO·Co_3_O_4_, peaks were observed at 2*θ* = 30.50°, 31.20°, 31.35°, 33.78°, 35.56°, 36.30°, 36.56°, 37.34°, 37.56°, 43.58°, 46.96°, 55.96°, 62.24°, and 64.92°, due to ZnO 100, 002, 101, 102, 110 and 103. Hexagonal ZnO with a lattice along the Co_3_O_4_ diffraction lines 111, 220, 313, 400, 422, 440, and 511 corresponded to a rectangular pyramidal crystal supported by JCPDS data (Fig. 1). Our results are in good agreement with previously reported results.^24^ No peaks signifying other combinations of cobalt were obtained, which confirms the purity and crystalline structure of the Co_3_O_4_ nanoparticle and ZnO·Co_3_O_4_. Additionally, the simulated XRD pattern of Co_3_O_4_ structure is in good agreement with the experimentally observed XRD pattern also indicated the purity of Co_3_O_4_ nanoparticles (Fig. 2). The size (∼15–31 nm) of the crystalline phase Co_3_O_4_ nanocrystal and the Zn porous crystal containing Co_3_O_4_ was calculated from the XRD data using the Scherrer equation (a).^25^ The similar range of the size of the particle was measured by TEM, which supports the stable phase of the crystalline material at room temperature after calcination.

### 3.4. EDX analysis

**Fig. 3:**
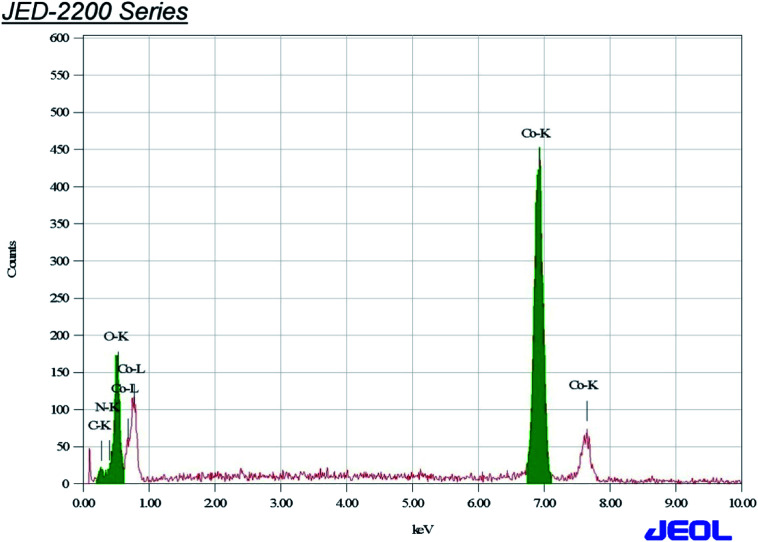
EDX of Co_3_O_4_.

**Fig. 4:**
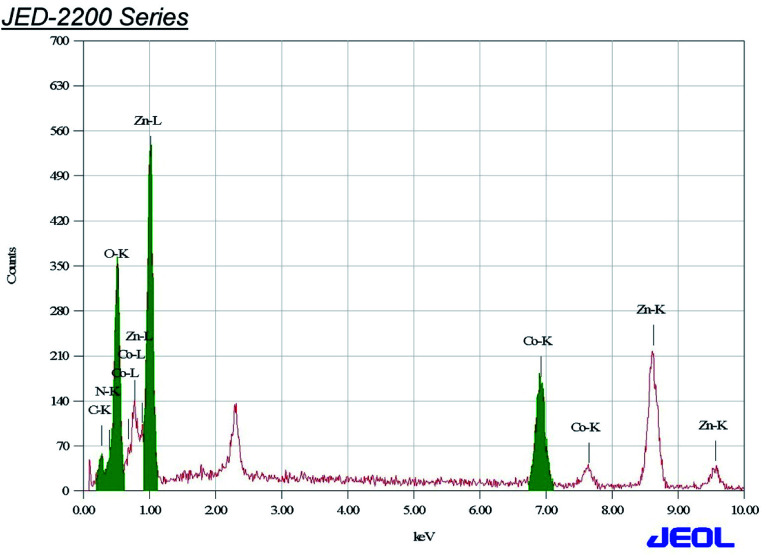
EDX of ZnO·Co_3_O_4_.

The Co_3_O_4_ and mixed oxide Zno·Co_3_O_4_ nanoparticles prepared by calcination of the [Co/Zn(ii)benzaldehyde·piperazine·H_2_O]complex at 500 °C were characterized by EDX, SEM and TEM. The analysis data supported the formation of one type of aggregated nanoparticles with an average size of 15–31 nm. The rectangular pyramidal morphology and size of the particles were further calculated by the XRD spectrum to validate the TEM results. Polycrystalline Co_3_O_4_ and porous ZnO·Co_3_O_4_ particles were analysed by energy-dispersive X-ray (EDX) spectra of Co_3_O_4_ (Fig. 3) and mixed ZnO·Co_3_O_4_ (Fig. 4). In the spectra, Co and O peaks were detected in both Co_3_O_4_ and ZnO·Co_3_O_4_, and Zn peaks were observed in the ZnO·Co_3_O_4_ spectra. This reveals that cobalt oxide aligned with ZnO to yield the composite. The EDX spectra of the oxides and element maps obtained showed the composition and mass percentages of ∼21% oxygen and 76% metals for the elements O, Co, and Zn, respectively. Carbon signals originating from grid background were observed at a negligible percentage.

### 3.5. Morphologies of CoCo_3_ and ZnO·Co_3_O_4_

**Fig. 5:**
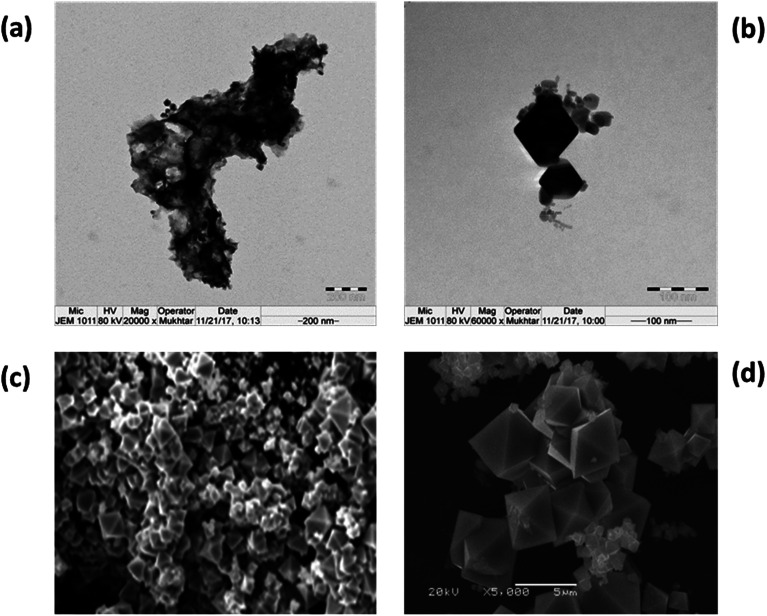
Typical TEM images: (a) Co_3_O_4_ nanoparticles and (b) the shape and size of the particles. Typical SEM images of a Co_3_O_4_ (c) bulk particle and (d) the geometry of Co_3_O_4_ particles.

**Fig. 6:**
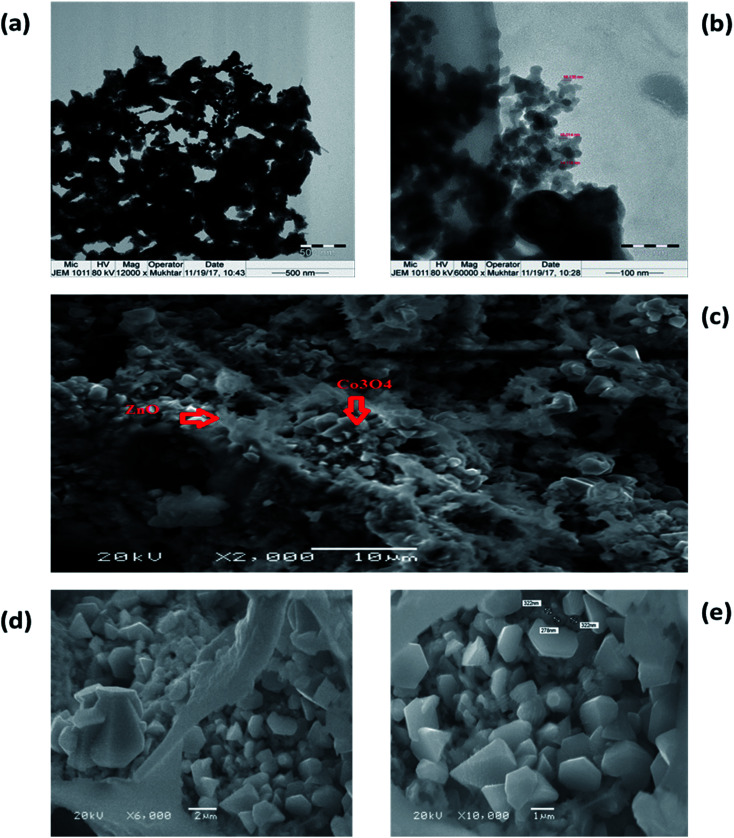
Typical TEM images, (a) and (b) of the structure and size of ZnO·Co_3_O_4_ particles. SEM images of ZnO·Co_3_O_4_, showing (c) the porous structure of ZnO with, (d) and (e), pockets of Co_3_O.

TEM and SEM images of nanomaterials prepared at 500 °C were obtained. The shape of the particles depends on the chemical environment precipitation and aggregation method. Homo and hetero metal oxides aggregated to minimize the interfacial energy are cubic nanoparticles with an average particle size of 15–31 nm (Fig. 5a–d). The morphology of Co_3_O_4_, with an average diameter of 31.0 nm, in a regular crystalline phase indicated that CoCo_3_ contains nanocrystals with a single shape. It was observed that the hexagonal crystalline porous ZnO cavity was uniformly filled with Co_3_O_4_ particles. The magnified SEM images show a uniform morphology (Fig. 6a–c). Porous hetero nanocrystals are important for electrochemical studies. To confirm the effect that the nature of the porous ZnO has on Co_3_O_4_ deposition, comparative cyclic voltammetry experiments were performed.

**Fig. 7:**
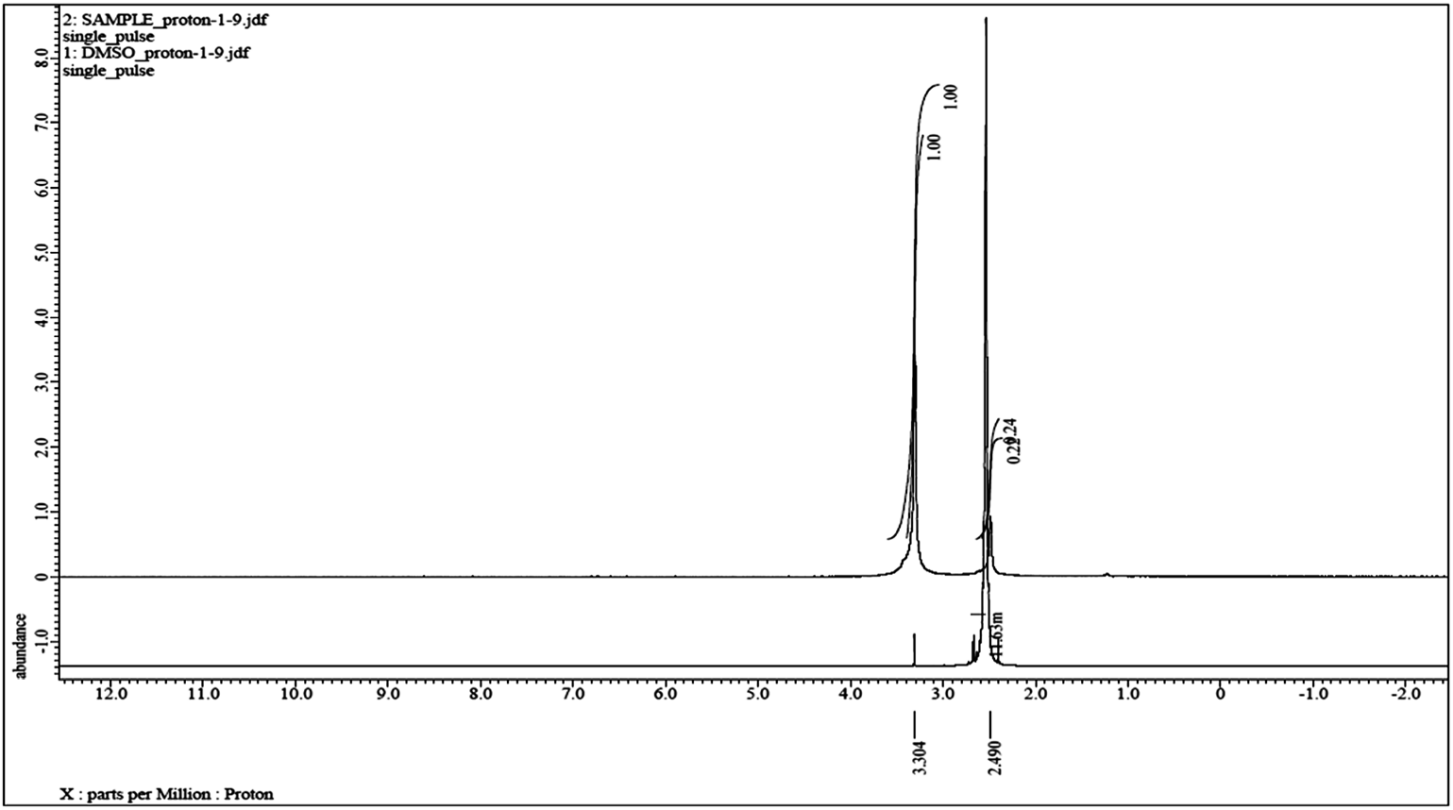
^1^H NMR chemical shift due to Co_3_O_4_.

**Fig. 8:**
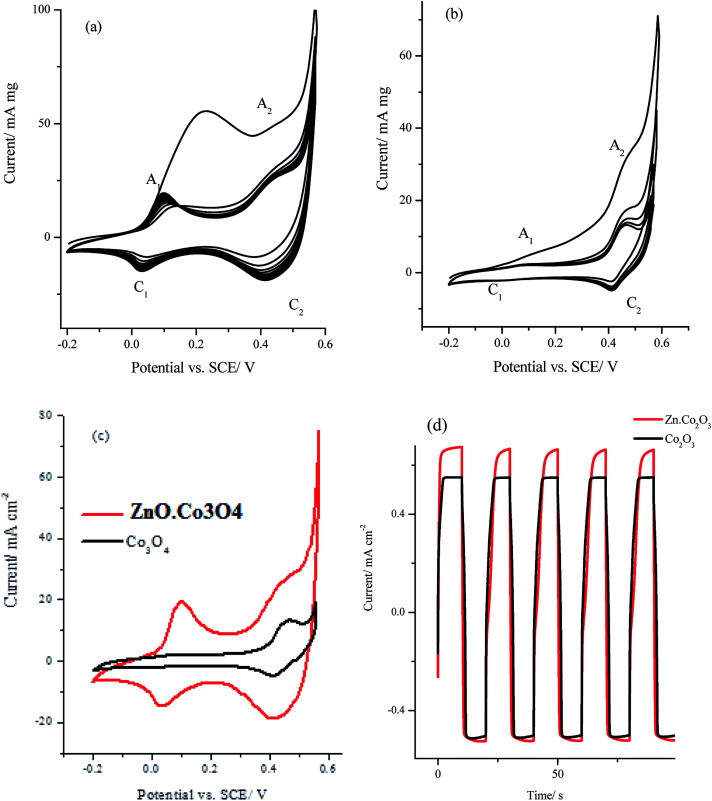
Multi-cyclic voltammetry at 50 mV s^−1^ in 1.0 M KOH for (a) for ZnO·Co_3_O_4_, (b) Co_3_O_4_ nanocrystals and (c) comparison for the stable cycle, (d) galvanostatic charge–discharge curves of ZnO·Co_3_O_4_ and Co_3_O_4_ electrodes at charge current of 4.0 A g^−1^.

**Fig. 9:**
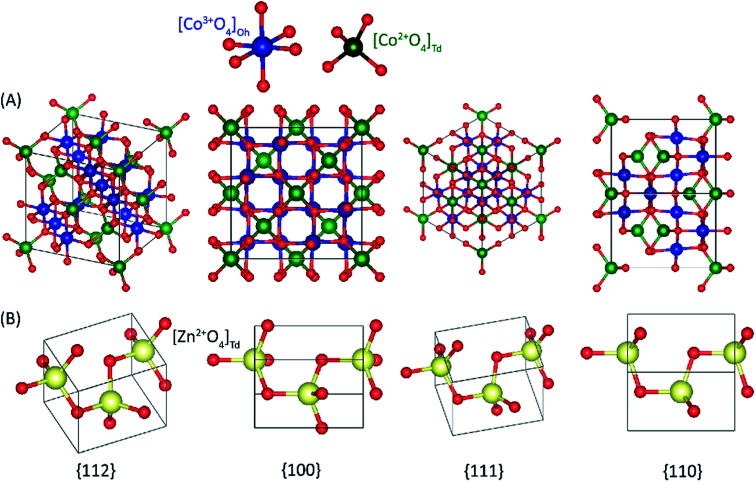
The illustration of atom configurations of the {100}, {110}, {111}, and {112} crystal planes of the Co_3_O_4_ spinel and ZnO structure.

**Fig. 10:**
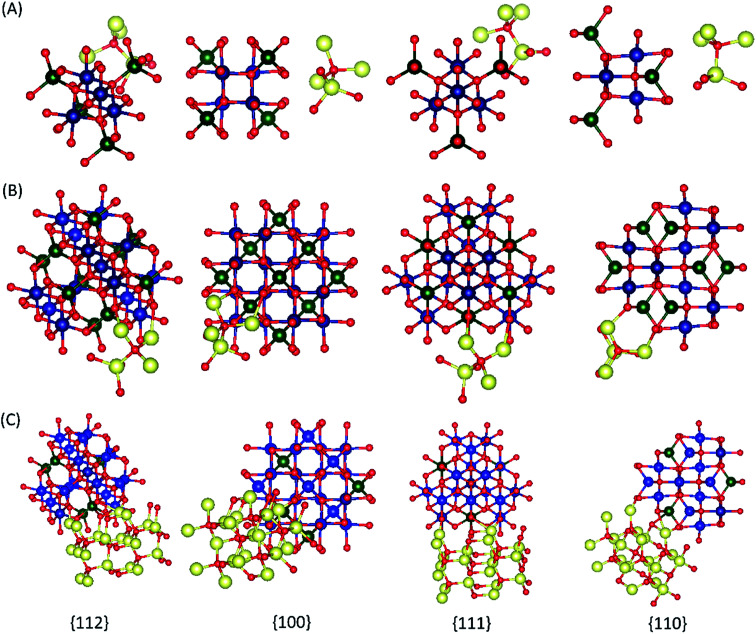
The illustration of atom configurations of the {100}, {110}, {111}, and {112} crystal planes of the global minimum energy geometry of Co_3_O_4_ZnO adducts, (A) model 1, (B) model 2, and (C) model 3.

Transmission electron microscopy (TEM) images of the homo and heteronano structure of Co_3_O_4_ and ZnO·Co_3_O_4_ (Fig. 5 and 6a, b) show low-magnification images of Co_3_O_4_ and mixed ZnO·Co_3_O_4_. The magnified images of Co_3_O_4_ exhibited a rectangular pyramidal type, as shown in Fig. 9a, with directional edges. The clear shape of the crystals was studied by SEM. The ZnO·Co_3_O_4_ material was scanned by TEM, and it was observed that the hetero nanostructure is a porous conjugate of mixed oxide. Fig. 10a and b also reveal nanopolycrystalline Co_3_O_4_ deposited in the single crystalline ZnO porous cavities. The TEM observation was further validated by SEM analysis (Fig. 6c and d).^26^

### 3.6. Paramagnetic character evaluation of nanoparticle Co_3_O_4_

NMR was employed to study the paramagnetic character of metal in nanophases in solution. The ^1^H NMR chemical shift due to Co_3_O_4_ was monitored by Evan's method. Evan's NMR studies^27,28^ showed a paramagnetic chemical shift of ∼0.8 ppm in the DMSO dimethyl proton signal at 2.49 ppm. The sharp change in the chemical shift at 3.3 ppm in the NMR spectrum clearly indicated that the particle exhibits paramagnetism (Fig. 7). The small shift in the NMR signals indicates that Co_3_O_4_ nanoparticles have weak ferromagnetic behaviour.

### 3.7. Electrochemistry

The cyclic voltammetry (CV) measurements for Co_3_O_4_ and ZnO·Co_3_O_4_ nanocrystals in 1.0 M KOH at a scan rate of 50 mV s^−1^ are depicted in Fig. 8. The cyclic voltammograms of the nanocrystals of Co_3_O_4_ and ZnO·Co_3_O_4_ (Fig. 8a and b) exhibited two redox peaks of (A_1_/C_1_) and (A_2_/C_2_) that could be attributed to the redox couples Co(ii)/Co(iii) and Co(iii)/Co(iv) and are located at the mid-peak potential of 65 and 430 mV *vs.* SCE, respectively.^29^ However, for the hetero-bimetallic nano oxide ZnO·Co_3_O_4_, the redox peaks were well resolved and had a significantly higher current than did the Co_3_O_4_ electrode as shown in Fig. 8c. Moreover, the oxidation current during the first anodic scan was much higher before it stabilized in the successive scans for both oxides, apparently due to a complete conversion of the ZnO·Co_3_O_4_ and Co_3_O_4_ to higher oxidized species, which was not completely reversible in the alkaline solution.^30–32^ The specific capacitance of the ZnO·Co_3_O_4_ and Co_3_O_4_ electrodes can be calculated from the CV using eqn (1):

1 *C*_s_ = *Q*/2*m*Δ*V*

where *C*_s_ is the specific capacitance in (F g^−1^), *Q* is the volumetric charge under the CV in coulomb, *m* is the mass of the oxide materials in grams and Δ*V* is the potential window of the cyclic voltammetry. Using the cyclic voltammetry in Fig. 8c, the specific capacitance values were estimated to be 207.3 and 51.1 F g^−1^ for ZnO·Co_3_O_4_ and Co_3_O_4_ electrodes, respectively. Moreover, Fig. 8d illustrates the galvanostatic charge–discharge curves of ZnO·Co_3_O_4_ and Co_3_O_4_ electrodes at charge current of 4.0 A g^−1^ and in 1.0 M KOH solution. The specific capacitance obtained from each discharge curve is equal 210.5 and 54.6 F g^−1^ for ZnO·Co_3_O_4_ and Co_3_O_4_ electrodes respectively which is in good agreement with the values obtained from the cyclic voltammetry. These observations indicate that heterometallic ZnO·Co_3_O_4_ exhibited a much higher specific capacitance than did a monometallic Co_3_O_4_ nanocrystal, which could be attributed presumably to the mesoporous structure and higher surface area that allow fast ion diffusion and better conductivity as a result of ZnO incorporation into the Co_3_O_4_ structure.^33^

### 3.8. Computational modelling

Ambient chemical transformations between nanoparticles of Co_3_O_4_ and ZnO leading to hybrid molecular adducts that preserve structure and topology are poorly explored area in material science. Atomically precise nanoparticles of Co and Zn metals, often called nanoclusters, which constitute an exploding discipline in nanomaterials because of their well-defined structures and drastic changes in their properties, in comparison to their bulk form, arising due to electronic confinement. Hence, theoretical calculations of ZnO·Co_3_O_4_ molecular aggregates have been performed in order to find out how closely they could approach each other and which atoms and their interactions would be involved in the adduct formation.

**Fig. 11:**
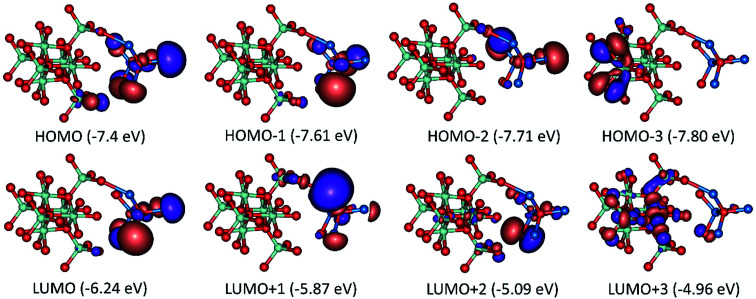
Frontier molecular orbitals and their energies of model 1: [Co_3_O_4_]_4_ [ZnO]_4_ adduct.

**Fig. 12:**
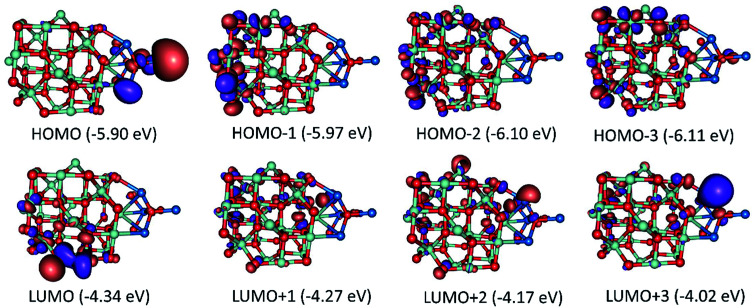
Frontier molecular orbitals and their energies of model 2: [Co_3_O_4_]_10_ [ZnO]_4_ adduct.

A complete search over the relevant rotational and translational degrees of freedom of Co_3_O_4_ nanocluster with respect to ZnO nanocluster in the Co_3_O_4_ZnO adduct with DFT is unfeasible due to the computational cost. Therefore, we used a combined approach utilizing the highly efficient force-field based method to identify a global minimum energy geometry of the Co_3_O_4_ZnO adducts, *e.g.* model 1: [Co_3_O_4_]_4_ [ZnO]_4_, model 2: [Co_3_O_4_]_10_ [ZnO]_4_, and model 3: [Co_3_O_4_]_10_ [ZnO]_20_ and then performed single point energy calculations using DFT method to calculate the electronic properties. We have used the reported crystal structure coordinates of Co_3_O_4_ and ZnO, without any structural relaxation, as the initial coordinates for geometry optimizations. The atom configurations of the {100}, {110}, {111}, and {112} crystal planes of the Co_3_O_4_ spinel and ZnO adducts are depicted in Fig. 9. Similarly, the crystal planes (100, 110, 111 and 112) of the global minimum energy geometries of the three adducts of Co_3_O_4_ and ZnO *e.g.* model 1: [Co_3_O_4_]_4_ [ZnO]_4_, model 2: [Co_3_O_4_]_10_ [ZnO]_4_, and model 3: [Co_3_O_4_]_10_ [ZnO]_20_ are illustrated in Fig. 10 and S5.† From the force-field global minimum geometries (FFGMG) of adducts, we identified that the significant changes are observed in the orientations and distances between the Co_3_O_4_ and ZnO adducts. In model 1, no bond formation observed and the nearest distance between the Co_3_O_4_ and ZnO adducts is found to be 2.527 Å (Zn⋯O). Whereas in model 2, Co_3_O_4_ and ZnO nanoclusters are approached so near to each other that they are linked together through the two Zn–O bonds (2.065 and 2.149 Å) between a bridging oxygen atom of [Co_3_O_4_]_10_ and a zinc atom of [ZnO]_4_ with Co–O–Zn oxo-linkage of 142.24° and 120° angles. Other two more close contacts with 2.24 Å also found between Zn and O atoms. Interestingly, in model 3, two different bonds, Zn–O: 1.935, 2.552 and 1.960 Å, between the zinc atoms of [ZnO]_20_ and oxygen atoms of [Co_3_O_4_]_10_; Co–O: 2.197 Å, between the cobalt atom of [Co_3_O_4_]_10_ and oxygen atom of [ZnO]_20_ are observed. The values of oxo-linkages angles Zn–O–Co: 144°, 149° and 111° and 94°: Co–O–Zn are observed. Additionally, a short O–O (1.463 Å) contact is also observed between the oxygen atoms of both adducts. Thus, from the computed structures of the adducts clearly suggested that as size of the interacted adducts increases more tightly they bound to each other *via* coordinate and covalent bonding and formed a single phase heterometallic nanomaterial. Further we have also demonstrated the corresponding frontier molecular orbitals of the model 1 and model 2, to explore the HOMO–LUMO gap and electronic properties (Fig. 11 and 12). The HOMO–LUMO gap is found to be 1.16 eV in model 1 while 1.56 eV in model 2. In the model 1, HOMO, HOMO−1, HOMO−2, LUMO, LUMO+1 and LUMO+2 are localized on the ZnO adduct while HOMO−3 and LUMO+3 on Co_3_O_4_ adduct (Fig. S5† and 11). Whereas in model 2, HOMO and LUMO are localized on the ZnO and Co_3_O_4_ adducts, respectively (Fig. 12).

**Fig. 13:**
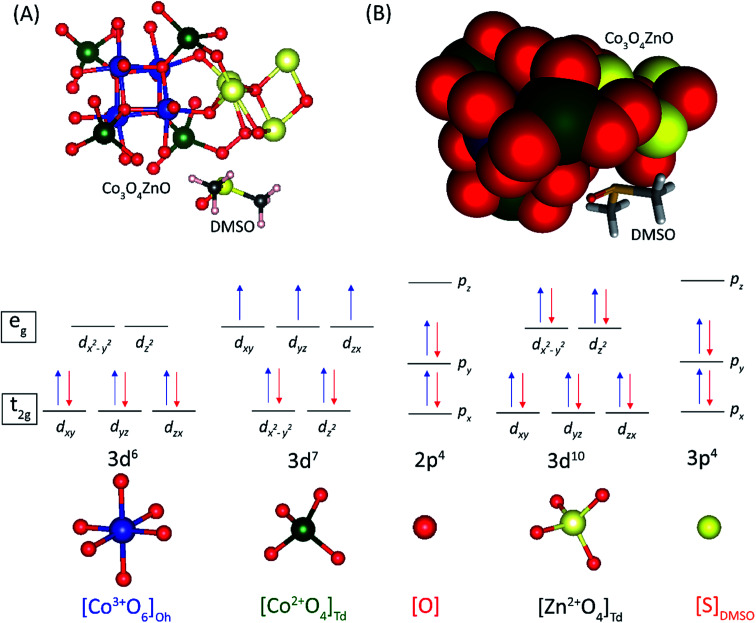
The schematic representation of crystal field diagram of the global minimum energy geometry of [Co_3_O_4_]_4_ [ZnO]_4_ DMSO adduct.

We have also performed the geometry optimization of model 1 with the DMSO molecule, to find out the paramagnetic nature of the Co_3_O_4_ZnO adduct which cause the shifting of ^1^H-NMR peaks of DMSO. The Co_3_O_4_ structure comprises a cubic close-packed array of O^−^ where 1/8 of the tetrahedral interstices are occupied by high-spin Co^2+^, whereas half of the octahedral interstices are occupied by low-spin Co^3+^. Each Co^2+^ (e_g_^4^ t_2g_^3^) is surrounded by four nearest neighbours of opposite spin, giving rise to an antiferromagnetic network. In contrast, the Co^3+^ exhibited a closed-shell configuration (t_2g_^6^) and nil magnetic moment, as depicted in Fig. 13. The global minimum energy geometry of model 1 with DMSO is illustrated in Fig. 13. The global minimum energy geometry indicated that in the presence of DMSO molecule the ZnO and Co_3_O_4_ adducts joined through the various Zn–O–Co linkage. Interestingly, Zn atom of ZnO adduct joined through the oxygen atoms of both types of Co^2+^ (*T*_d_) and Co^3+^ (*O*_h_) ions of Co_3_O_4_ adduct (Fig. 13). Such kind of linkage may perturbed the antiferromagnetic coupling between the Co^2+^ (*T*_d_) ions or ligand field stabilization, which cause the paramagnetic nature of the Co_3_O_4_ZnO adduct and this paramagnetism cause the shifting of ^1^H-NMR peaks of DMSO as observed experimentally.

## 4. Conclusion

The present method for preparing nanoparticle precursors for homo- and heterometallic cores is more superior to other methods because we have used Werner's coordination theory reaction and reaction environment. One type of the nanocrystal was obtained, in comparison to other methods, which produce a mixture of nanoparticles with different shapes and sizes. This method is simple and suitable for nanocrystal design and tailoring. Co_3_O_4_ nanoparticles can be produced at a low temperature in the absence of solvent, surfactant and expensive or complicated equipment. The pure nanostructure Co_3_O_4_ and ZnO·Co_3_O_4_ particles with a size of 18–20 nm was successfully synthesized by the thermal decomposition of [M(ii)L,L′] (where M = Co(ii), Zn(ii) L= 4-hydroxy benzaldehyde and L′ = piperazine) complexes as new precursors. Nanoparticles were formed by redox reactions among the piperazine, benzaldehyde and counter ions. The rectangular/cubic rhomboid Co_3_O_4_ nanoparticles were obtained with agglomeration. DFT calculations support the formation and structure of the ZnO·Co_3_O_4_ and Co_3_O_4_. The model has been generated to understand the nano structures. Weak ferromagnetic behaviour was observed in the NMR chemical shift of methyl proton signals. The specific capacitance of the ZnO·Co_3_O_4_ and Co_3_O_4_ electrodes was calculated from the CV; ZnO·Co_3_O_4_ exhibited high capacitance which attributed to the better conductivity and surface area. DFT calculations with DMSO molecule further validated the experimental results displaying the paramagnetic nature of the Co(ii) ion in Co_3_O_4_ adduct which caused the shifting of ^1^H-NMR peaks of DMSO.

## Conflicts of interest

There is no conflicts to declare.

## Supplementary Material

RA-010-D0RA01191F-s001
