# Supplementary material for: Synthesis of homo- and hetero-metallic cobalt and zinc nano oxide particles by a calcination process using coordination compounds: their characterization, DFT calculations and capacitance behavioural study
Source: RSC Adv. 2020 Apr 1;10(22):13126–38. doi: 10.1039/d0ra01191f (PMC9051467; doi:10.1039/d0ra01191f)
Supplement: RA-010-D0RA01191F-s001 [file RA-010-D0RA01191F-s001.pdf]

Electronic Supplementary Material (ESI) for RSC Advances.
This journal is © The Royal Society of Chemistry 2020

SI data -RA-ART-02-2020-001191

Synthesis of homo- and hetero-metallic cobalt and zinc nano oxide particles by a calcination process using coordination compounds: Their characterization, DFT calculations and capacitance behavioural study

Sartaj Tabassum, [φ, $,*], Mohammad Usman[φ], Hamad A. Al-Lohedan[$,] Mahmood M. S. Abdull[$,], Mohamed A. Ghanem,[$] Merfat S. Al-Sharif[$], Mohd Sajid Ali[$] .

[φ] *Department of Chemistry, Aligarh Muslim University, Aligarh-202002, India*

[$]*Department of Chemistry, College of Sciences, King Saud University, P.O. Box 2455, Riyadh 11451, KSA.*

**corresponding author, email: tsartaj62@yahoo.com, +919358255791.*

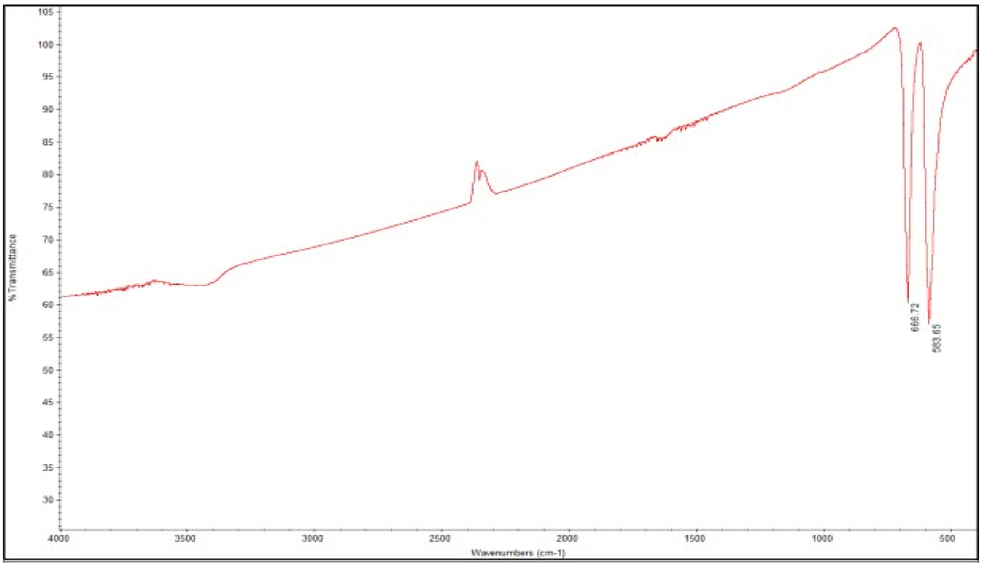

Fig.S1 FTIR spectrum of Co_3O_4 nanoparticles.

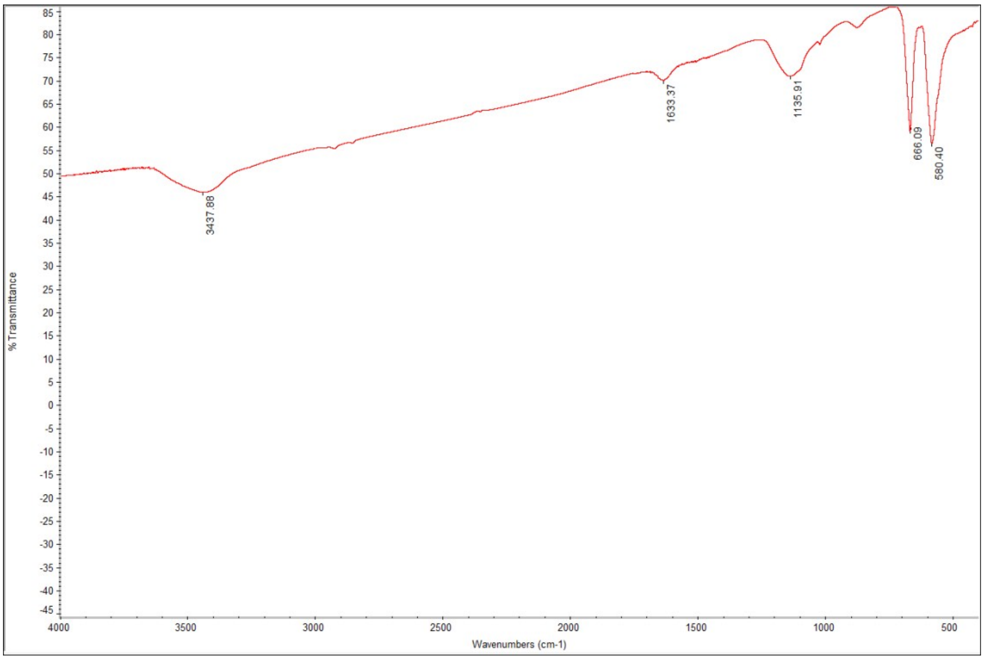

Fig. S2 FTIR spectrum of $ZnOCo_3O_4$.

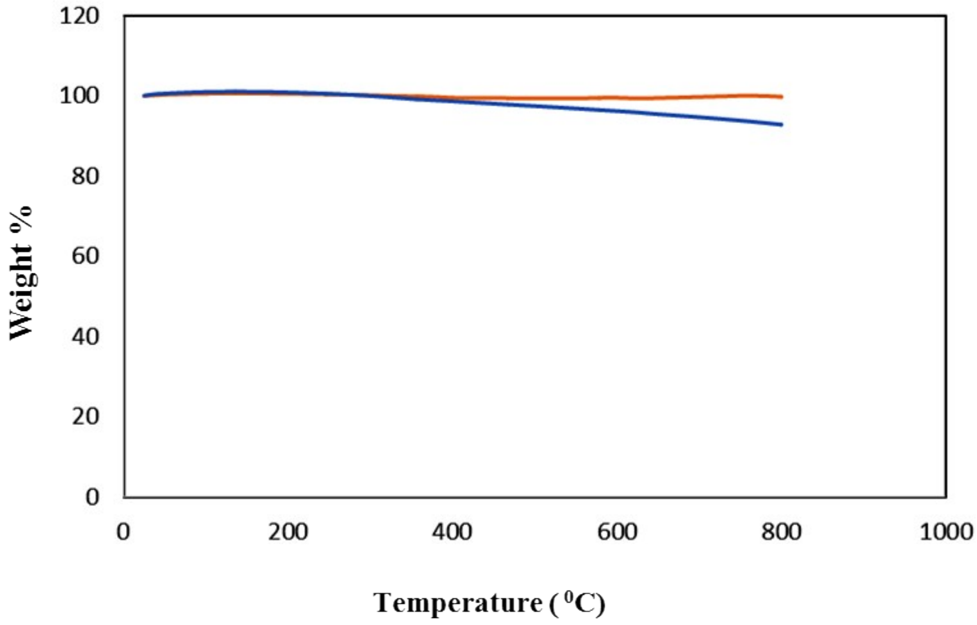

Fig. S3 TGA of the nanoparticles.

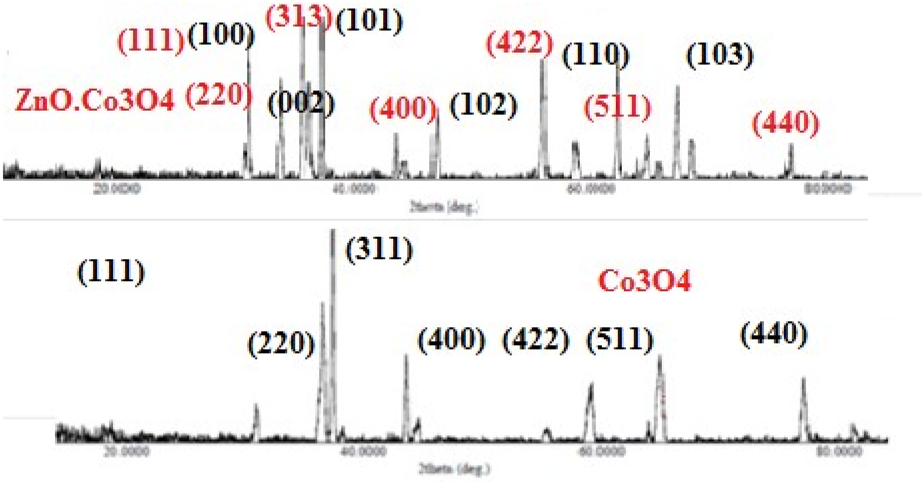

Fig. S4 XRD pattern line with lattice parameters ZnO.Co_3O_4 and Co_3O_4. (JCPDS Data Card No: 36-1451)

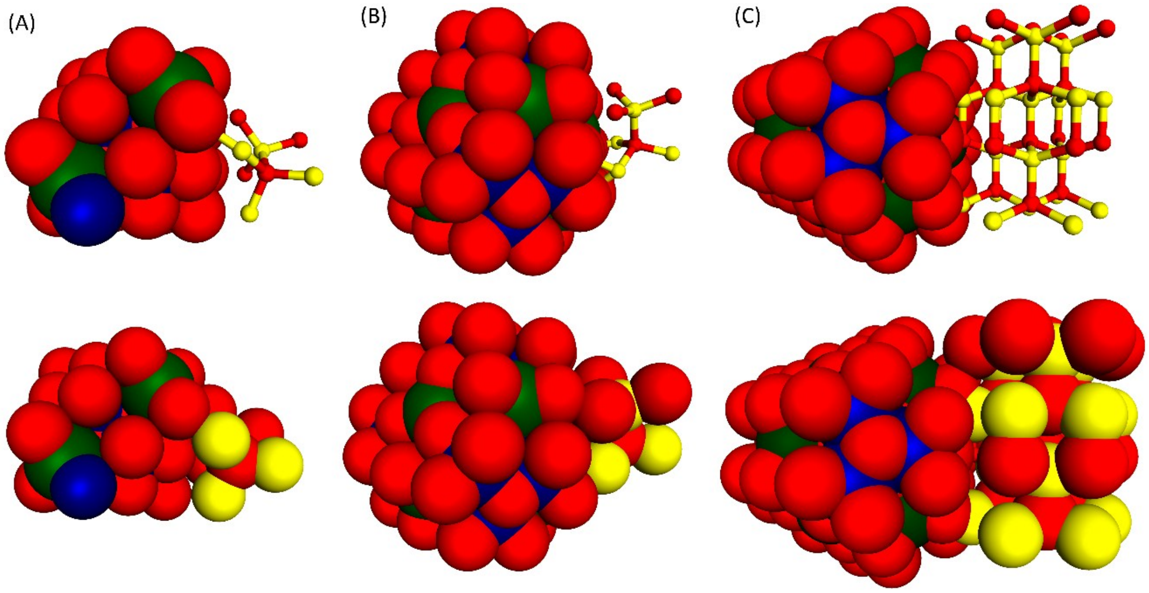

Fig. S5. The Illustration of CPK models of the global minimum energy geometry of Co3O4ZnO adducts, (A) Model 1, (B) Model 2, and (C) Model 3.
